# Growth-stage-related shifts in diatom endometabolome composition set the stage for bacterial heterotrophy

**DOI:** 10.1038/s43705-022-00116-5

**Published:** 2022-03-30

**Authors:** Malin Olofsson, Frank X. Ferrer-González, Mario Uchimiya, Jeremy E. Schreier, Nicole R. Holderman, Christa B. Smith, Arthur S. Edison, Mary Ann Moran

**Affiliations:** 1 Department of Marine Sciences, University of Georgia, Athens, GA 30602 USA; 2 Department of Aquatic Sciences and Assessment, Swedish University of Agricultural Sciences, 750 57 Uppsala, Sweden; 3 Department of Biochemistry and Complex Carbohydrate Research Center, University of Georgia, Athens, GA 30602 USA

**Keywords:** Biogeochemistry, Ecology

## Abstract

Phytoplankton-derived metabolites fuel a large fraction of heterotrophic bacterial production in the global ocean, yet methodological challenges have limited our understanding of the organic molecules transferred between these microbial groups. In an experimental bloom study consisting of three heterotrophic marine bacteria growing together with the diatom *Thalassiosira pseudonana*, we concurrently measured diatom endometabolites (i.e., potential exometabolite supply) by nuclear magnetic resonance (NMR) spectroscopy and bacterial gene expression (i.e., potential exometabolite uptake) by metatranscriptomic sequencing. Twenty-two diatom endometabolites were annotated, with nine increasing in internal concentration in the late stage of the bloom, eight decreasing, and five showing no variation through the bloom progression. Some metabolite changes could be linked to shifts in diatom gene expression, as well as to shifts in bacterial community composition and their expression of substrate uptake and catabolism genes. Yet an overall low match indicated that endometabolome concentration was not a good predictor of exometabolite availability, and that complex physiological and ecological interactions underlie metabolite exchange. Six diatom endometabolites accumulated to higher concentrations in the bacterial co-cultures compared to axenic cultures, suggesting a bacterial influence on rates of synthesis or release of glutamate, arginine, leucine, 2,3-dihydroxypropane-1-sulfonate, glucose, and glycerol-3-phosphate. Better understanding of phytoplankton metabolite production, release, and transfer to assembled bacterial communities is key to untangling this nearly invisible yet pivotal step in ocean carbon cycling.

## Introduction

Phytoplankton bloom development and senescence are closely entangled with heterotrophic bacterial community activities, mediated through phytoplankton-derived dissolved organic carbon [1–3]. Newly released phytoplankton metabolites can be rapidly consumed by bacterial assemblages, often within minutes to days [4]. As marine phytoplankton are responsible for half of Earth’s photosynthesis and much of the fixed carbon is passed on to heterotrophic bacteria, quantifying this step is important for modeling global carbon flux [5–7].

Metabolite pools derived from phytoplankton consist of hundreds of unique organic compounds, most of which accumulate in only trace amounts [8]. This diversity coupled to high turnover rates poses a challenge for chemical identification, with only 1–5% of compounds identified thus far [8–10]. Transfer of these metabolites to bacteria has been even more difficult to quantify. Recent studies have made headway by analysis of bacterial gene expression as an indication of uptake and catabolism of substrates [11–14], yet information is lacking on the diversity of roles of bacterial community members in determining exometabolite flux and fate.

The extent and composition of direct release of phytoplankton photosynthate is influenced by the physiological state of the cell [15–17]. Healthy phytoplankton cells release labile compounds such as sugars, sugar alcohols, amino acids, and carboxylic acids [2, 18, 19], which may dominate during early phases of a bloom. As blooms progress towards senescence, the amount of metabolites released increases, and larger molecules such as polysaccharides take on more importance [2, 20, 21]. This release of labile carbon from phytoplankton to surrounding organisms can occur by multiple mechanisms [22]. The simplest is diffusion between intracellular pools and external seawater [23], although this is constrained to molecules of relatively small size [24]. Alternatively, molecules can be actively released via overflow pathways when rates of photosynthesis exceed their needs for growth, for example, carbohydrates are released in response to photorespiration [18, 23, 25]. Active release of metabolites can also occur in response to associated microbes [3, 26], such as through the release of molecules that serve as bacterial chemoattractants [27, 28].

Substrate release sets the stage for bacterial heterotrophy, with different substrate preferences governing the succession of taxa during a bloom [19]. Among heterotrophic bacterial taxa consistently found associated with phytoplankton there is evidence for specialization on certain components of extracellular release [2, 19], even when the extracellular release originates from a single phytoplankton species [14]. The ability to use common but distinct substrate sets is likely a benefit to these bacterial groups, contributing to their success in surface ocean communities [2, 29]. Detailed understanding of endometabolite composition, release, and utilization by heterotrophic bacteria under different stages of phytoplankton growth is still limited, however, particularly when there are multiple bacterial species that can compete and interact.

Globally, primary production is strongly influenced by annual spring blooms in temperate regions, commonly dominated by fast-growing diatoms [19, 30]. To mimic a diatom bloom under controlled laboratory conditions, *Thalassiosira pseudonana* was co-cultured with a synthetic community consisting of three heterotrophic bacteria (*Ruegeria pomeroyi*, *Stenotrophomonas* sp., and *Polaribacter dokdonensis*) representing taxa typically associated with phytoplankton blooms [19]. We used nuclear magnetic resonance (NMR) spectroscopy to identify the endometabolites of the diatom and transcriptomics to trace their potential transfer to bacteria during early and late bloom stages. Coupling diatom endometabolite quantification with bacterial gene expression analysis also enabled us to observe temporal patterns, either matched or mismatched, that are potentially informative of extracellular release mechanisms.

## Methods

### Co-culture conditions

During this synthetic bloom experiment, axenic cultures of the diatom *T. pseudonana* CCMP1335 (National Center for Marine Algae) were inoculated with equal cell numbers (~3 x 10^4^ cells ml^−1^) of the heterotrophic bacteria *R. pomeroyi* DSS-3 (Rhodobacterales; ATCC 700808; isolated from southeastern US seawater [31]), *Stenotrophomonas* sp. SKA14 (Xanthomonadales; provided by J. Pinhassi, Linnaeus University Sweden; isolated from the Skagerrak Sea [32]) and *P. dokdonensis* MED152 (Flavobacteriales; provided by J. Pinhassi, Linnaeus University Sweden; isolated from the Mediterranean Sea [33]). The strains have high 16S rRNA gene identity to bacteria associated with phytoplankton cultures or flow-sorted with phytoplankton cells, with percent similarities up to 99.6% for *R. pomeroyi* [3, 34–36], 98.8% for *Stenotrophomonas* sp. SKA14 [34], and 97.2% for *P. dokdonensis* [35, 37]. The diatom was grown in organic carbon-free L1 medium [38] prepared in acid-washed glass containers at a salinity of 35 [39] for one week prior to the start of the experiment. Cultures were grown with a 16:8 h light:dark cycle under 160 µmol photons m^−2^ s^−1^ at 18 °C and checked for bacterial contamination by plating on rich medium (YTSS). On day 0 of the experiment, diatoms were transferred into 1.9 L culture flasks containing 1 L of medium to a final concentration of ~2 × 10^3^ cells ml^-1^. The medium was made with ^13^C-bicarbonate (Cambridge Isotope Libraries, Inc., Tewksbury, MA, USA) to enhance NMR signals. One flask was kept as L1 medium without organisms for use as a background control for NMR analyses.

The three strains of heterotrophic bacteria were grown overnight in either YTSS at 30 °C (*R. pomeroyi* and *Stenotrophomonas*) or ^1^/5YTSS at 25 °C (*P. dokdonensis*) made with salinity 20 artificial seawater. Cells were harvested in exponential growth phase and washed five times in the same artificial seawater used for preparing the L1 medium (1 ml wash volume). The bacteria were inoculated in equal proportions of OD_600_ into 15 flasks containing diatoms, with a final combined concentration of ~1 × 10^5^ cells ml^−1^, which is comparable to pre-bloom conditions during a natural bloom [19]. One set of three flasks remained axenic. Three co-culture flasks were sacrificed after 8 h of light (day 0), and then on days 3, 8, 15, 20. The axenic flasks were only sampled on day 15.

To trace diatom and bacterial growth, 1 ml subsamples were fixed with glutaraldehyde (1% final concentration), stored overnight at 4 °C, and thereafter at −80 °C until flow cytometric analysis. The samples were stained with SYBR® Green I (final concentration 1×; Life Technologies, Carlsbad, CA, USA) and analyzed on a CyAn ADP flow cytometer (Beckman Coulter, Hialeah, Florida) using 5-µm fluorescent particles (Spherotech, Lake Forest, IL, USA) for enumeration. The diatom specific growth rate (μ d^−1^) was calculated as (ln *D*_E_ − ln *D*_S_)/(*t*_E_ − *t*_S_), where *D*_E_ is cell number at the end of a period and *D*_S_ at the start of the experiment, and *t*_E_ is the end day and *t*_S_ the start day. The three morphologically distinct bacteria (*P. dokdonensis* by orange color; *Stenotrophomonas* sp. by fast growth and large colonies; *R. pomeroyi* by slower growth and small colonies; Fig. S1) were individually quantified as colony-forming units (CFUs) by dilution plating. Subsamples of 100 µl were diluted 10^−1^ to 10^−7^ times and spread onto both YTSS and ^1^/5YTSS agar plates. The plates were incubated at 30 °C (YTSS) or 25 °C (^1^/5YTSS) and counted after three and four days, respectively. Total bacterial cell numbers measured by flow cytometry correlated well with the sum of the species-specific CFUs (Fig. S1).

For sampling diatoms, subsamples (100–200 ml for RNA and 700–1000 ml for endometabolites) were gently filtered using a peristaltic pump onto 2.0 µm pore-size polycarbonate Isopore^TM^ filters (Millipore, Burlington, MA, USA). For sampling bacteria, the filtrate from the endometabolite samples was re-filtered onto 0.2 µm pore-size Supor^®^ PES filters (PALL, Port Washington, NY, USA). Filters collected for diatom and bacterial RNA were immediately flash-frozen in liquid nitrogen, and along with endometabolite filters transferred to −80 °C until processing. Subsamples of the final filtrate were collected for dissolved inorganic nutrient analysis (10 ml stored at −20 °C).

### Diatom endometabolite analysis

For endometabolite analysis (day 3 and 15), filters in tubes were sonicated for 7 min while submerged in ice-water to remove diatom cells (50 s on and 10 s off sequence) using an SLPe sonifier (Branson, Brookfield, CT, USA) after adding 15 ml of ultrapure water, and the liquid fraction was collected in fresh tubes as described in Uchimiya et al. [40]. This process was repeated three times and combined fractions stored at −80 °C until processing. Samples were lyophilized (Labconco, Kansas City, MO, USA) and pellets were mixed with 600 µL of phosphate buffer (30 mM phosphate in deuterated water, pH 7.4) and 1 mM internal standard (2, 2-dimethyl-2-silapentane-5-sulfonate). Samples were vortexed for 5 min, centrifuged at 20 800 RCF for 10 min, and supernatants were transferred to 5 mm NMR tubes (Bruker, Billerica, MA, USA). One pooled quality control sample was prepared by combining aliquots of all the samples and used for annotation. All sample processing was carried out at 4 °C. Metabolites were analyzed by NMR spectroscopy using a 600 MHz AVANCE III HD instrument (Bruker) equipped with a 5 mm TXI probe and pulse programs of ^1^H-^13^C heteronuclear single quantum correlation (HSQC, hsqcetgpprsisp2.2 by Bruker nomenclature) and ^1^H-^13^C HSQC-total correlation spectroscopy (HSQC-TOCSY, hsqcdietgpsisp.2). Data were processed by TopSpin version 4.0 (Bruker). Peak intensity was extracted by rNMR version 1.11 [41], normalized by cell number and auto-scaled. Metabolites were annotated based on chemical shift (HSQC) and spin network information (HSQC-TOCSY) (Fig. S2). Chemical shift values for candidate peaks were obtained from Biological Magnetic Resonance Data Bank [42], and raw HSQC spectra for validation from Human Metabolome Databases [43]. Four compounds of interest that are not in these databases were annotated using literature values (homarine, [44]; 2,3-dihydroxypropane-1-sulfonate (DHPS), dimethylsulfoniopropionate (DMSP), and β-1,3-glucan, [17]). A confidence level of annotation was assigned to each metabolite, where 1 = putative compounds with functional group information; 2 = partially matched to HSQC chemical shift information in the databases or literature; 3 = fully matched to HSQC chemical shift; 4 = fully matched to HSQC chemical shift and validated by HSQC-TOCSY; 5 = validated by a spiking experiment. All the data, sample preparation protocols, and NMR analysis and processing parameters are deposited in Metabolomics Workbench under Project ID 001231 (10.21228/M8KT3K). Data were converted to Z-scores (value – mean/standard deviation). Statistical analysis of day 3 vs day 15 co-cultures, and day 15 co-cultures versus day 15 axenic cultures was conducted by using unpaired *T*-tests (*p* ≤ 0.05, *n* = 3).

### RNA extraction

RNA was extracted using the ZymoBIOMICS RNA Miniprep Kit (Zymo Research, Irvine, CA, USA) according to the manufacturer’s protocol with 20 min beating and on-column DNase treatment. Following extraction, an additional DNA removal was performed using the TURBO DNA-*free* Kit (Invitrogen, ThermoFisher Scientific, Vilnius, Lithuania) following standard kit procedures. Stranded RNA libraries were prepared using the Zymo-Seq RiboFree Total RNA Library Kit (ZymoBIOMICS) with rRNA depletion. Libraries and rRNA depletion for samples with low RNA concentration were prepared at HudsonAlpha Discovery (Huntsville, AL, USA) and all libraries were sequenced on the Illumina NextSeq platform (SE, 75 bp).

### RNA-Seq and differential gene expression analyses

The TrimGalore toolkit was used for sequence trimming and quality control, imposing a minimum quality score of 20. Reads aligning to the rRNA sequences of the microbial taxa were removed using SortMeRNA. STAR aligner was used to map remaining reads to the genome of each of the species and HTSeq to count reads mapped to each gene. Genes with differential expression between co-culture time points (day 3 and 15) and between co-culture and axenic conditions (day 15; diatoms only) were identified using DESeq2 in R (Version 4.0.0). Biosynthesis pathways were identified based on Biocyc [45] and PhyloDB [46]. Heatmaps were created using the pheatmap package in R.

RNA-Seq data from a previous study [14] that was collected for each bacterial strain when in individual co-culture with the diatom was compared to the day 3 RNA-Seq data from this study. The datasets were analyzed using DESeq2 as described above, with each co-culture compared to the same reference dataset [14], and significantly enriched genes emerging from the analyses were compared. The reference dataset was established by growing bacteria in the same medium as used for both co-cultures, except that *T. pseudonana* was not inoculated and 2.5 mM glucose was added [14]; this provided transcriptomes of actively growing bacteria on a defined carbon source against which the two co-culture datasets were analyzed.

### Dissolved inorganic nutrients

Dissolved inorganic nutrient concentrations were measured at day 15 in both axenic and co-cultures as well as in the media-only control (Table S1). Nitrate and nitrite were measured by the automated cadmium reduction method, phosphate by the automated ascorbic acid reduction method, and ammonium by the automated phenate method [47]. Pure water was used as a blank and 2 ml samples were analyzed using standard curves of 0, 50, 100, 200 ppb on a Alpkem RFA 300 (UniGreenScheme, UK). Silicate was analyzed spectrophotometrically [48, 49] (1 ml sample + 4 ml water; Spectronic 301; Milton Roy, Ivyland, PA, USA) using a standard curve composed of 0, 5, 10, 20, and 40 μM solutions and a water blank processed through same chemistry as the samples.

## Results and discussion

### Co-culture dynamics

**Fig. 1:**
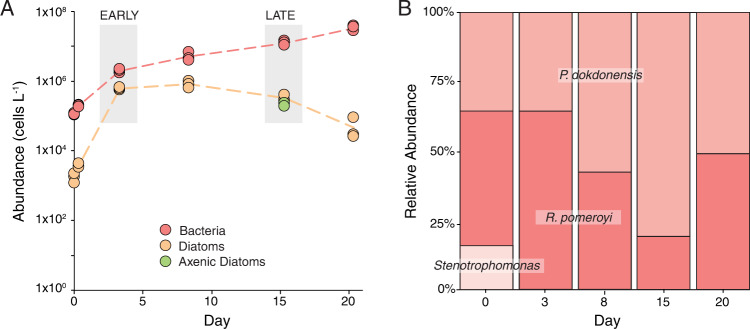
Time course of microbial abundances. **A** Cell abundance based on flow cytometric analysis for co-cultures (5 time points) and axenic cultures (day 15 only) (*n* = 3). The intensive sampling dates for the early and late bloom comparisons are marked with gray boxes. **B** Mean relative abundance of bacterial species is based on CFUs (*n* = 3). The day 0 samples were collected 8 h after inoculation.

This study was designed to enhance understanding of metabolite release and utilization across bloom stages in a simple community of phytoplankton and heterotrophic bacteria. The synthetic community was established with the diatom *T. pseudonana* and the bacterial strains *R. pomeroyi* DSS-3, *Stenotrophomonas* sp. SKA14, and *P. dokdonensis* MED152. These bacterial strains have high genetic similarity to isolates from phytoplankton cultures [14] and represent taxa that are common in phytoplankton blooms. Metabolites derived from the diatom were the sole source of carbon available for the bacteria, since no organic substrates were added. In addition, none of the bacteria can assimilate nitrate, and usable nitrogen was only available as diatom or bacterial extracellular products. The diatom had its highest specific growth rate of 1.65 d^−1^ during days 0–3, after which the rate declined (Fig. 1A). The total abundance of heterotrophic bacteria increased steadily but there was a succession that favored *P. dokdonensis* through day 15, and then *R. pomeroyi* by day 20; *Stenotrophomonas* disappeared from the model system by day 3 (Fig. 1B). The presence of bacteria did not affect the growth of diatoms based on comparisons of abundance in co-cultures versus axenic cultures at day 15 (Fig. 1A), as has been found previously [14, 26]. Inorganic nutrients were not limiting (>5 μM at day 15; Table S1).

### Diatom endometabolite shifts

**Fig. 2:**
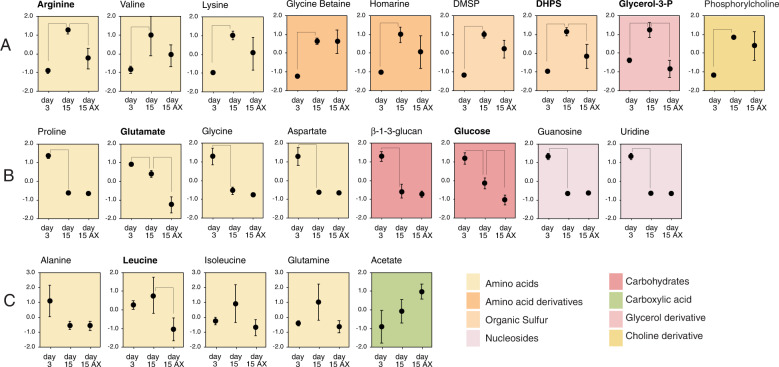
Relative endometabolite abundance in diatom cells. Abundance is expressed as mean *Z*-score of per cell concentration in early bloom co-cultures (day 3), late bloom co-cultures (day 15), and axenic late bloom cultures (day 15 AX). Metabolites present in significantly different per cell concentrations are linked by brackets (*T*-test, *p* ≤ 0.05, *n* = 3); no statistical comparisons were made between day 3 and day 15 AX. Row **A** Endometabolites with significantly higher concentration in day 15 co-cultures compared to day 3 co-cultures; Row **B** Endometabolites with significantly higher concentration in day 3 co-cultures compared to day 15 co-cultures; Row **C** Endometabolites not significantly different between day 3 and day 15. Bold font highlights the metabolites accumulating to higher concentrations in 15 d co-cultures compared to 15 d axenic cultures. Plots are colored according to metabolite class. Error bars represent standard deviations. See Table S3 for metabolite intensity per cell.

Analyses focused on the day 3 (early bloom) and day 15 (late bloom) co-culture time points, for which a complete set of metabolomic and transcriptomic data were collected. Twenty-two diatom endometabolites that were annotated with high confidence by NMR analysis (Table S2) and quantified after normalizing to diatom cell number revealed that endometabolome composition differed substantially between bloom stages. Metabolites with significantly different cellular concentrations included nine compounds that were higher in intracellular concentration during the late bloom; these were arginine, valine, lysine, DHPS, glycerol-3-phosphate, phosphorylcholine, DMSP, glycine betaine, and homarine (*T*-test; *P* < 0.05, *n* = 3; Fig. 2A; Table S3), of which the last three are known to function as osmolytes [50, 51]. Elevated internal DMSP concentrations in phytoplankton cells have been linked to nitrogen, CO_2_, silicate, and phosphate limitation, increasing during stationary growth phase and potentially replacing nitrogen-rich osmolytes or serving as an antioxidant under stressful conditions such as CO_2_ limitation and low temperature [52]. All three osmolytes have also been identified in endometabolomes of natural plankton communities from surface seawater [53]. Eight metabolites were significantly lower in intracellular concentration in the late bloom co-cultures; these were proline, glutamate, glycine, β-1,3-glucan, aspartate, glucose, guanosine, and uridine (Fig. 2B). Five metabolites did not change between early and late bloom stages; these were alanine, leucine, isoleucine, glutamine, and acetate (Fig. 2C).

Differences in diatom endometabolome composition were also evident in comparisons of the 15 d cultures with and without a bacterial community. Six diatom endometabolites had accumulated to higher concentrations in the presence of bacteria compared to axenic cultures by day 15; these were glutamate, arginine, leucine, DHPS, glucose, and glycerol-3-phosphate; none accumulated to higher concentrations in axenic cultures (Fig. 2), similar to previous analyses [17]. Alteration of phytoplankton physiology in the presence of bacteria has been reported previously and attributed to processes such as bacterial remineralization of ammonium from dissolved organic matter [54] and release of vitamins [13] or hormones [3]. In this study, phytoplankton were both nutrient and vitamin replete and there was no evidence that co-culturing with bacteria enhanced growth. Nonetheless, the diatom cells accumulated endometabolites differently depending on the presence or absence of bacteria.

### Diatom gene expression

**Fig. 3:**
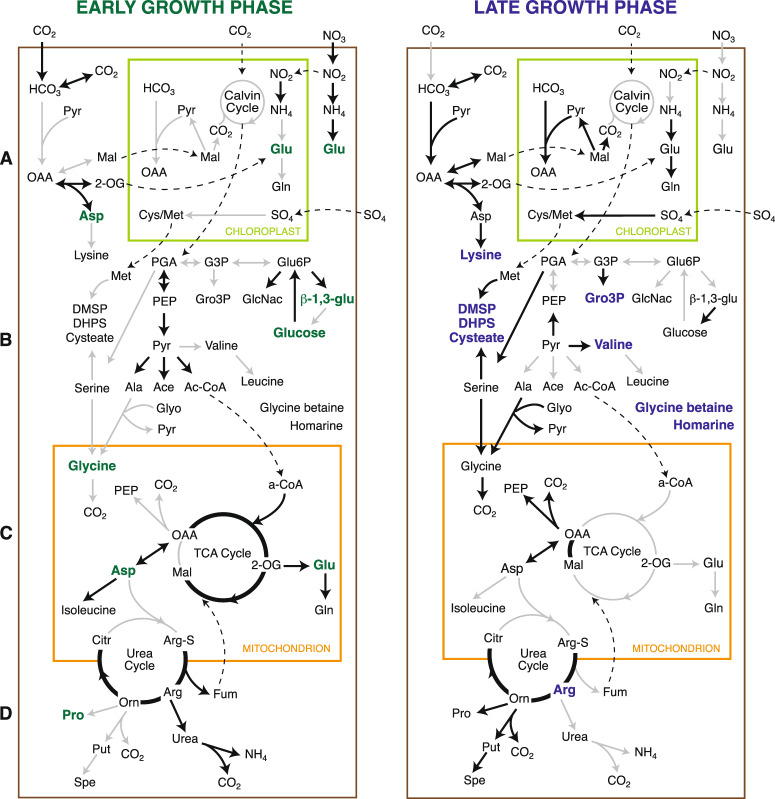
Integration of diatom endometabolite and gene expression data for early-stage (left panel) and late-stage (right panel) bloom phases. Black lines indicate relative gene expression that is significantly higher in one growth stage compared to the other, gray lines indicate expression that is not significantly higher. Green font indicates significantly higher metabolite concentration in early-stage cells, and blue font indicates higher concentration in late-stage cells (see Fig. 2). **A** Carbon and nitrogen assimilation. **B** Glycolysis/Gluconeogenesis. **C** TCA cycle. **D** Urea cycle. Metabolite abbreviations are as follows: Ala alanine, Ace acetate, Ac-CoA acetyl-CoA, Arg arginine, β-1,3-glu β-1,3-glucan, Citr citrulline, Cyst cysteate, Arg-S Arginine-succinate, Fum fumarate, GlcNac *N*-acetyl-D-glucosamine, Glu glutamate, Gln glutamine, Glyo glyoxylate, Mal malate, G3P glyceraldehyde-3-phosphate, Gro3P glycerol-3-phosphate, 2-OG oxoglutarate, OAA oxaloacetate, PEP phosphoenolpyruvate, Orn ornithine, Pyr pyruvate, PGA phosphoglycerate, Put putrescine, Spe spermidine.

Diatom gene expression provided insights into physiological changes associated with growth stage. Relative gene expression fell into two distinct expression clusters consisting of early (days 0, 3) and late (days 8, 15, 20) sample times (Fig. S3). Comparing the day 3 and day 15 time points, 6 637 of the 11 675 predicted genes in the *T. pseudonana* genome (59%) had significantly different relative contributions to the transcriptome. The diatom’s early bloom transcriptome was highly enriched in transcripts for CO_2_ acquisition via carbonic anhydrases (Fig. 3A). Transcripts for synthesis of glycolysis products pyruvate, acetate, and acetyl-CoA (Fig. 3B) and for channeling acetyl-CoA into the tricarboxylic acid (TCA) cycle were also enriched, as were transcripts for multiple central TCA cycle steps from oxaloacetate to succinate (Fig. 3C). Transcription patterns indicated that recently fixed carbon was directed toward chitin precursor *N*-acetyl-D-glucosamine (GlcNAc) and chrysolaminarin backbone β-1,3-glucan (Table S4). These transcriptome features were consistent with the higher concentrations of glucose and β-1,3-glucan in the diatom endometabolome on day 3. Overall, this gene expression pattern indicated an emphasis on carbon fixation and biomass building in the early bloom (Fig. 2) and is consistent with the diatom’s highest specific growth rate occurring during days 0–3 (Fig. 1).

Early-stage transcript enrichment was also observed for genes mediating nitrate assimilation and conversion to ammonium. Glutamate is a central component of biosynthesis pathways for amino acids and the assimilation pathway for ammonium, which serves as the nitrogen homeostasis mechanism. Compared to late phase *T. pseudonana* cells, glutamate synthesis genes were enriched in both the cytosol and mitochondrion (Fig. 3A, B and Table S4). Indeed, higher internal endometabolome concentrations of glutamate as well as other nitrogen-rich metabolites (amino acids proline, glycine, and aspartate; and nucleosides guanosine and uridine) were evident in the early growth stage diatoms (Fig. 2). *T. pseudonana*’s urea cycle coordinates cellular nitrogen and carbon status [55], and several genes in this cycle were enriched during early growth (Fig. 3D). These patterns are consistent with peak concentrations of nitrogen-containing amino acids and nucleosides in the early bloom, and together indicate higher nitrogen requirements by *T. pseudonana* during the biomass building phase.

Diatom transcripts in the late bloom phase were enriched instead with genes that synthesize malate and oxaloacetate, two metabolites of the C_4_ delivery pathway for CO_2_ that performs well under low CO_2_ concentrations (Fig. 3A). Whether C_4_ metabolism is functional in diatoms is controversial (e.g., refs [56–58]) but if so, it would be beneficial in cases where inorganic carbon concentrations decrease in late phase blooms. Late-phase enriched transcripts were also found in pathways for putrescine and spermidine synthesis. These polyamines have several known functions in diatom cells, among them a role in stress response [59]. In field studies, seawater concentrations of polyamines increased during diatom bloom decline [60]. Evidence for physiological stress on late bloom diatoms also included increased expression of genes for synthesis of the osmolyte DMSP (Fig. 3); the diatom pathways for synthesizing osmolytes glycine betaine and homarine are unknown.

Genes related to sulfate assimilation and subsequent synthesis of the organic sulfur compounds cysteate, sulfopyruvate, sulfolactate, and DHPS, in addition to DMSP mentioned above, were highly enriched in the later stage cultures, with up to 100-fold increases in relative expression (Fig. 3B and Table S4). These data are congruent with higher DMSP and DHPS endometabolite concentrations in late- compared to early-stage diatom cells (Fig. 2). The temporal switch from greater investments in synthesis of nitrogen-rich compounds in the early-stage bloom to organic sulfur compounds in the late stage could reflect the higher energy costs for nitrate assimilation compared to sulfate assimilation (41 versus 33 ATP per mol assimilated; [61]). Similar to nitrate transformation to nitrite and ammonium, sulfate transformation to cysteate also consumes NADPH, and thus removes excess reductants produced during photosynthesis [62]. Genes for cysteate catabolism, believed to be an intermediate in DHPS formation [50], were significantly enriched during the late growth phase. Relatively higher sulfate usage as compared to nitrogen in aging diatom cultures or during bloom senescence is generally associated with nitrate limitation [62], but here we observed this even under nitrate replete conditions, suggesting a physiological response by the cells as their growth rate slowed. Giordano and Raven [61] suggest that low early ocean sulfate concentrations may have influenced the evolution of marine phytoplankton, manifested in divergent present-day strategies for regulation of nitrogen versus sulfur metabolism.

### Bacterial gene expression

The early versus late bloom differences evident in the diatom metabolome and transcriptome set the stage for responses by associated heterotrophic bacteria in their substrate acquisition patterns. The substrates available to the co-cultured bacteria were inferred from significant differences in relative expression of genes diagnostic for uptake of organic matter (i.e., those mediating transport or initial catabolism of exogenous molecules). We focus on *R. pomeroyi* and *P. dokdonensis* in this analysis as *Stenotrophomonas* sp. was not present in the co-cultures at day 15 (Fig. 1B).

Expression patterns of *R. pomeroyi* genes suggested higher relative availability of taurine, glycerol-3-phosphate, lactate, DHPS, putrescine, alanine, and glycine betaine in the early bloom; and of *N*-acetyltaurine, glycine, choline, spermidine, glycolate, trimethylamine, and sugars during late bloom (Tables 1 and S5). Transcription patterns suggested that DMSP was available at both time points but degraded by different pathways in the early (demethylation pathway) versus late (cleavage pathway) bloom [63], a temporal switch in pathway dominance consistent with previous studies in cultures [26, 64] and in a natural phytoplankton bloom [65], and potentially driven by differences in reactive oxygen stress between pathways [65]. Enriched bacterial gene expression for spermidine transport coincided with enriched diatom gene expression for its biosynthesis. Overall, however, there were few positive relationships between shifts in *R. pomeroyi* gene expression for uptake of a metabolite and shifts in phytoplankton intracellular concentration or biosynthetic gene expression for that same metabolite. For example, of eleven metabolites found in the diatom endometabolome and for which the *R. pomeroyi* transporters are known, we found that glycine betaine, DHPS, and glycerol-3-phosphate had higher concentrations in phytoplankton cells on day 15 but their transporters were enriched in the bacterial transcriptome on day 3; glycine had higher concentrations in phytoplankton cells on day 3 but the transporter was enriched on day 15; alanine and acetate did not differ in concentration between early versus late bloom phytoplankton cells but their transporters were enriched on day 3 and 15, respectively; and proline, aspartate, glucose, glutamate, and phosphorylcholine were either more abundant in day 3 or day 15 phytoplankton cells but their transporter expression in *R. pomeroyi* was not different. Assuming bacterial transporter expression is induced primarily by substrate availability [66], these mismatches argue for a minor role for passive leakage in metabolite release and instead support active release mechanisms that cannot be predicted from endometabolite concentrations. An additional ten metabolites detected in the phytoplankton metabolome do not have confirmed transporters in the *R. pomeroyi* genome: lysine, glutamine, arginine, valine, leucine, isoleucine, homarine, guanosine, uridine, and β-1,3-glucan.

**Table 1:** Genes from *R. pomeroyi* (top) and *P. dokdonensis* (bottom) with relevance to diatom endometabolome composition and with significantly higher relative expression (*p* ≤ 0.05) during early (left) or late (right) diatom growth phase.

Early growth phase		Late growth phase	
Function	Locus tag	Function	Locus tag
*Ruegeria pomeroyi*			
Glycine betaine transporter	SPO2441	*N*-acetyltaurine transporter	SPO0661
DMSP – demethylation	SPO0677, 3804 3805	DMSP – cleavage	SPO1703
Taurine transporter	SPO0674, 0676	Glycine	SPOA0310
Putrescine transporter	SPO3473, 3474	Choline	SPO1083, 0084
DHPS degradation	SPO0158	Spermidine transporter	SPO3467
Lactic acid transporter	SPO1017-1021	Glycolate	SPOA0143
Glycerol-3-phosphate transporter	SPO0239, 0240	Glycerol transporter	SPO0608-0612
Alanine symporter	SPO2370	Trimethylamine transporter	SPO1551, 1552, 1562
Amino acid transporter	SPO1516	Sugar transporter	SPO0608-0612
		Acetate	SPO2963
*Polaribacter dokdonensis*			
Hypothetical protein ^PUL2^	MED152_00445	MFS transporter (fucose) ^PUL3^	MED152_05095
Glycosyl hydrolase family 17 ^PUL2^	MED152_00450	MFS transporter (fucose) ^PUL3^	MED152_05115
O-glycosyl hydrolase family 30 ^PUL2^	MED152_00455	MFS transporter (maltose) ^PUL3^	MED152_05120
MFS transporter (glycoside etc. family) ^PUL2^	MED152_00460	AraC family transcriptional regulator ^PUL3^	MED152_05185
Choline dehydrogenase ^PUL3^	MED152_05190	Sugar kinase ^PUL3^	MED152_05080
Sugar transporter (glucose/galactose) ^PUL5^	MED152_08460	Alginate lyase	MED152_06195
DNA-binding response regulator ^PUL5^	MED152_08465	Sugar transporter	MED152_09090
Histidine kinase ^PUL5^	MED152_08470	Polysaccharide transporter	MED152_03130
SusC/RagA family TonB-linked outer membrane protein ^PUL5^	MED152_08475	Glyceraldehyde-3-P dehydrogenase	MED152_13444
SusD/RagB family nutrient binding outer membrane lipoprotein ^PUL5^	MED152_08480		
Glucosamine-6-phosphate deaminase ^PUL5^	MED152_08485		
CAZyme; beta-N-acetylhexosaminidase (GH20) ^PUL5^	MED152_08490		
N-acetylglucosaminase kinase (GlcNac) ^PUL5^	MED152_08495		
Glycosyl hydrolase 81 (GH81)	MED152_00755		
Glycogen synthase (GT4)	MED152_05855		
Glycine cleavage	MED152_06700		
1,4-α-glucan branching enzyme	MED152_05875		
Glyceraldehyde-3-P dehydrogenase	MED152_03295		

^PUL2^Chrysolaminarin uptake; ^PUL3^Unknown/multiple function; ^PUL5^*N*-acetyl-D-glucosamine uptake.

Flavobacteriia member *P. dokdonensis* increased in abundance through time in the co-cultures, as has been observed for marine flavobacteria in natural diatom bloom progression [19]. This pattern has been attributed to specialization by this taxon for glycan utilization, release of which increases in aging phytoplankton cells [67]. Gene expression patterns by *P. dokdonensis* were also consistent with increasing glycan importance in the late bloom, particularly for genes active in polysaccharide utilization loci (PULs) [68]. These genomic regions enable hydrolysis of polysaccharides to monomers, which are transported into the periplasm and subsequently transported across the cell membrane. Several genes within PUL3 (as designated in the CAZy database; Taxonomy ID 313598 [14, 69]) were significantly enriched during the late growth phase, including two annotated as fucose permeases (Table 1 and S6). *Polaribacter* species were shown to peak concurrently with fucosidase activity in a natural bloom [19], indicating an association with fucose degradation within the genus. Genes in PUL5 hypothesized to transport and degrade chitin subunits [14] exhibited up to 40-fold enrichment in late bloom *P. dokdonensis* transcriptomes. Chitin is a common organic molecule in the ocean [70–72], and serves as component of cell walls and a locus for bacterial attachment in diatoms [73, 74].

In addition to utilization of structural polysaccharides, gene expression in *P. dokdonensis* PUL2 suggested utilization of the storage polysaccharide chrysolaminarin ([14]; Table 1). This parallels the high transcriptional signal and endometabolite concentration for the β-1,3-glucan building block of chrysolaminarin in early-stage *T. pseudonana* (Fig. 3B and Table S4) [75, 76]. Phytoplankton were recently shown to exhibit daily cycles of internal chrysolaminarin concentrations synchronized with diel light patterns [17, 76], with this storage polysaccharide comprising up to 80% of diatom carbon under certain conditions [75]. The utilization of both structural and storage polysaccharides represented a distinct niche for *P. dokdonensis* in the *T. pseudonana* co-cultures. As found previously [14], *R. pomeroyi* and *P. dokdonensis* transcriptomes indicated little overlap in resource use, with each species responding to different suites of organic compounds in early and late bloom stages.

There was temporal matching of peaks in diatom endometabolite concentrations and *P. dokdonensis* gene expression for early bloom peaks of proline, glucose, and β-1,3-glucan. Of these, proline is a candidate for passive diffusion because of its small size, but the higher molecular weight metabolites glucose and β-1,3-glucan are less likely to diffuse through the diatom membrane, suggesting instead a link to export for physiological balance [18, 23, 25, 77]. Previous studies have found glucose release from diatoms when carbon fixation rates are high [78], likely due to photosynthetic overflow pathways. Yet the poor match between peaks in diatom endometabolites and peaks in both *P. dokdonensis* and *R. pomeroyi* transcription indicate that endometabolite abundance cannot predict the release of metabolites into surrounding seawater (Table S2), and argues for a key role for active (i.e., non-diffusive) mechanisms of diatom metabolite release. Also of interest are the six metabolites (arginine, DHPS, glycerol-3-phosphate, glutamate, glucose, and leucine) with higher concentrations in phytoplankton cells in co-culture compared to axenic culture (Fig. 2). Although the mechanism is unclear, bacteria appeared to directly or indirectly affect the internal concentrations of these metabolites.

### Community vs. individual bacterial gene expression

These same three bacterial species were co-cultured individually with *T. pseudonana* in a prior study [14], providing the opportunity to explore gene expression as a member of a bacterial community versus when alone. Several conditions differed between the studies; in Ferrer-González et al. [14], exometabolites built up in axenic *T. pseudonana* cultures for 7 days prior to inoculation of the bacteria, followed by sampling after 8 h. In the present study, the diatom and bacteria were inoculated at the same time, with sampling after 3 d. In both studies, however, the diatom was in exponential growth at the time of bacterial sampling. Significantly enriched bacterial genes were identified based on a reference dataset collected from bacteria growing in a glucose medium. Of the 24 enriched transporters in *R. pomeroyi*, 12 were enriched only in the individual co-cultures, including those with experimentally-verified substrates *N*-acetyltaurine and urea; and four were enriched only in the community co-cultures, including those with experimentally-verified substrates choline, acetate, and DMSP (Table S7). There was no difference in distribution of the three dominant compound classes represented by the transporters (organic acids, amino acids, and organic sulfur compounds), nor in the distribution of nitrogenous compound transporters (Table S7). For *P. dokdonensis*, the enrichment of genes in PULs 2, 3, 5, 6, and 7 seen in this study matched the Ferrer-González study [14]. However, PUL4 (no annotation available) had only one enriched gene matching and PUL8 (annotated for siderophore uptake and potentially colicin toxins [79]) had none (Table S8). Because the study designs differed in factors other than just the number of bacterial species, these comparisons are viewed as preliminary. Nonetheless, they suggest differences in uptake when bacteria are members of a community, with potential mechanisms including altered metabolite release by the diatom, or antagonism or competition for resources by the bacteria.

## Conclusions

The interaction between phytoplankton and bacteria in the surface ocean represents a central biogeochemical relationship that is relevant at the global scale. In a model bloom experiment with the marine diatom *T. pseudonana* providing the only source of organic matter to a heterotrophic bacterial community, we characterized potential substrates and considered their mechanisms of release. The diatom’s endometabolome was dynamic, differed with bloom stage, and was affected by the presence of bacteria. The diatom’s transcriptome similarly differed between bloom stages, changing from a composition enabling carbon fixation and nitrogen metabolism regulation to one focused on organic sulfur compound synthesis. Bacterial transporter expression suggested that metabolite availability differed between early and late bloom, and that the bacterial species maintained distinct resource niches through the bloom. The dynamics of bacterial uptake system expression matched the dynamics of endometabolite concentrations in just a few cases, specifically for proline, glucose, and β-1,3-glucan. The majority of molecules, however, did not have synchronous patterns, suggesting complex interaction scenarios that reflect both phytoplankton physiology and bacterial influence.

## Supplementary information

Supplementary Information

Supplementary Table S3

Supplementary Table S4

Supplementary Table S5

Supplementary Table S6

## Data Availability

Transcriptome data and associated metadata are deposited at NCBI under BioProject ID PRJNA758094. Metabolomics data and associated sample preparation protocols and NMR analysis and processing parameters are deposited at the Metabolomics Workbench Data Repository under Project ID 001231 (10.21228/M8KT3K). Additional data products and metadata are available at GitHub (10.5281/zenodo.6344452).
