# Supplementary material for: Growth-stage-related shifts in diatom endometabolome composition set the stage for bacterial heterotrophy
Source: ISME Commun. 2022 Mar 30;2:28. doi: 10.1038/s43705-022-00116-5 (PMC9723723; doi:10.1038/s43705-022-00116-5)
Supplement: Supplementary file 1 — Supplementary Information [file 43705_2022_116_MOESM1_ESM.pdf]

Supplementary Information:

Growth-Stage Related Shifts in Diatom Endometabolome Composition Set the Stage for Bacterial Heterotrophy

Malin Olofsson[1,2], Frank X. Ferrer-González[1], Mario Uchimiya[1,3], Jeremy E. Schreier[1], Nicole R. Holderman[3], Christa B. Smith[1], Arthur S. Edison[3], Mary Ann Moran[1]

[1]Department of Marine Sciences, University of Georgia, Athens, GA 30602, USA

[2]Department of Aquatic Sciences and Assessment, Swedish University of Agricultural Sciences, 750 57 Uppsala, Sweden

[3]Department of Biochemistry and Complex Carbohydrate Research Center, University of Georgia, Athens, GA 30602, USA

*Corresponding author: Mary Ann Moran, mmoran@uga.edu, 706-542-6481

Contents:
Supplementary Figures S1, S2, S3
Supplementary Tables S1, S2

As Separate Excel Files:
Supplementary Tables S3, S4, S5, S6

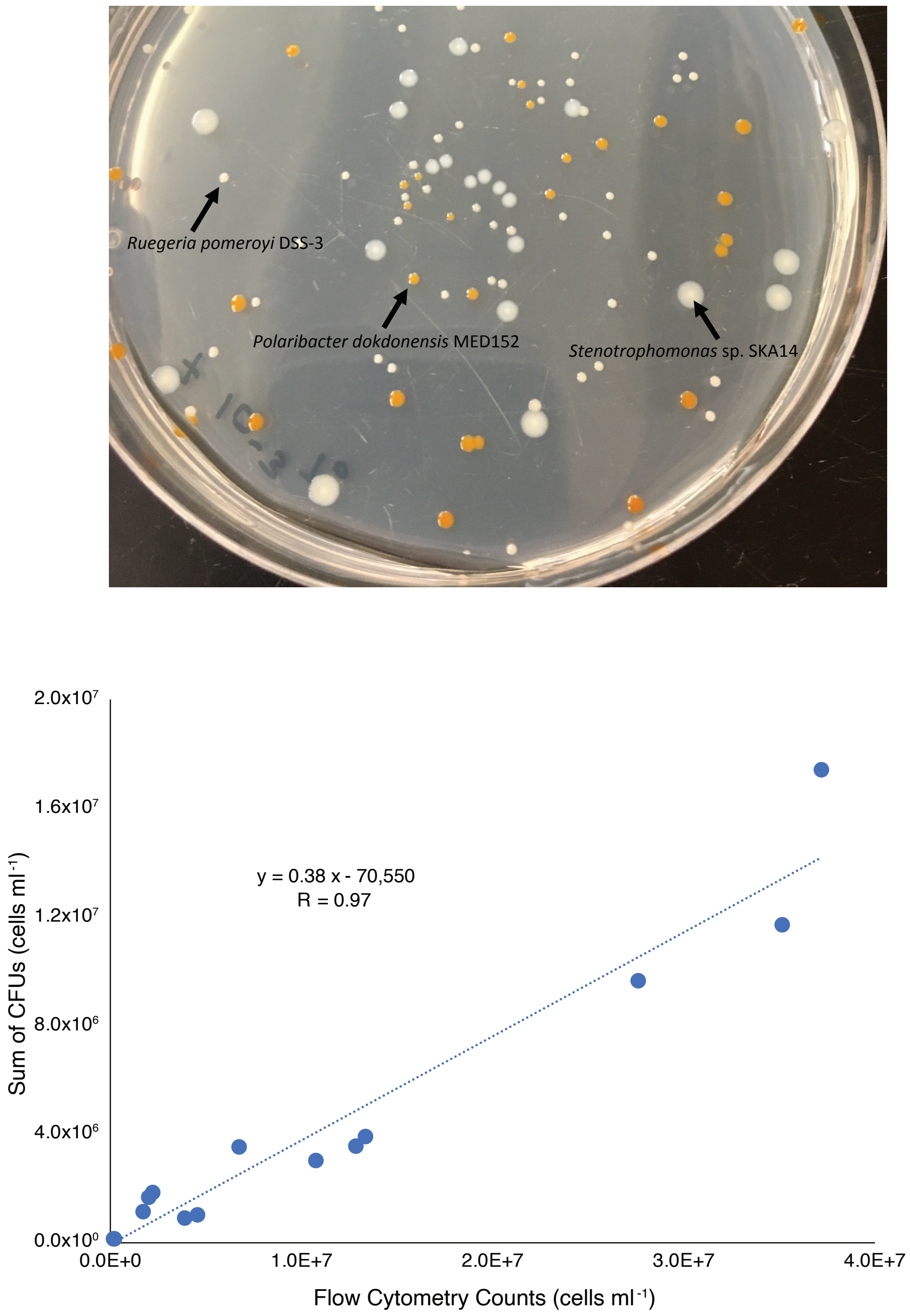

Figure S1. Top: For CFU analysis, bacterial species were distinguished based on colony morhpology on 1/2 YTSS agar plates. Bottom: CFUs for the three bacteria were summed and compared to total bacterial cell counts obtained from flow cytometry. The two measures were significantly correlated, with the sum of CFUs 2.4-fold lower than flow cytometery counts.

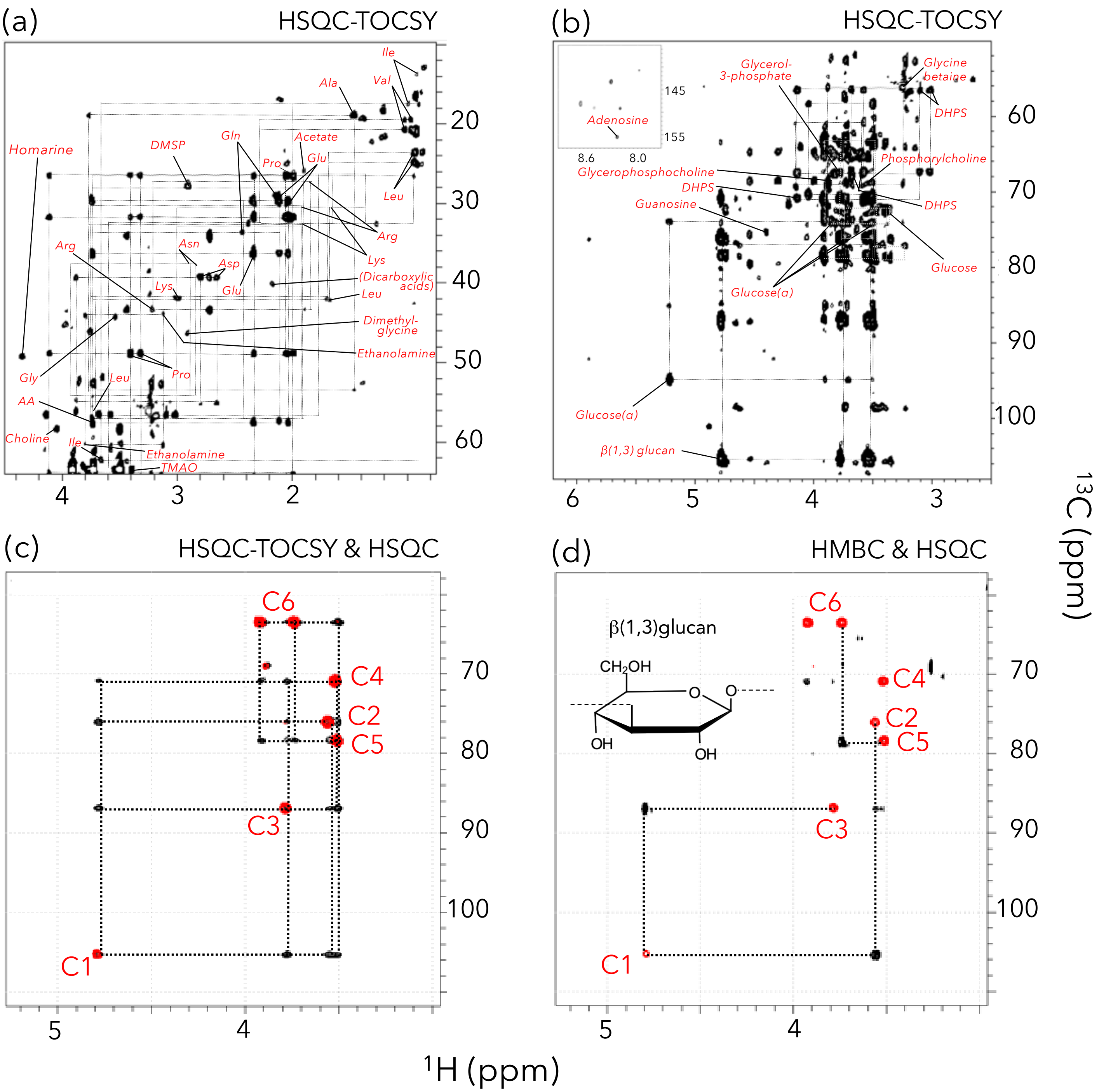

Figure S2. Diatom endometabolome annotation methods. Representative peak(s) for compounds indicated on HSQC-TOCSY spectra (a and b), additional structural validation (e.g., for polysaccharide β-1,3-glucan) by HSQC-TOCSY (c), and HMBC experiments (d). Peaks from HSQC experiments are overlaid and colored in red. A complete compound list is provided in Table 1, and chemical shift information used for annotation is provided in Table S1. 3-Hydroxybutyrate, 4-hydroxyphenylacetate, and uridine are not visible in (a) and (b) due to relatively low intensities. AA, amino acid alpha carbon.

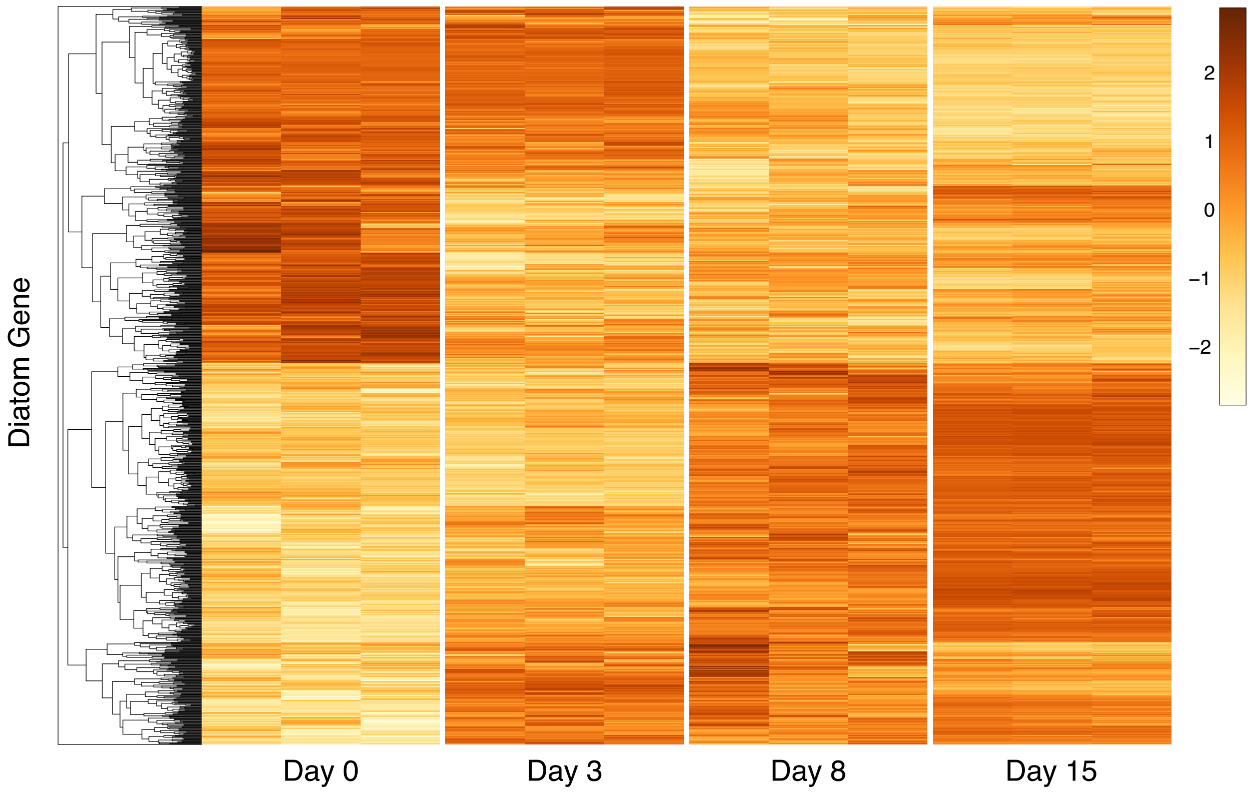

Figure S3. Relative gene expression of the 1000 *T. pseudonana* genes with the largest changes across sample points. Transcription patterns clustered by sample day and temporal sequence. Each replicate is shown individually, and values are given as *Z*-scores.

Table S1. Dissolved inorganic nutrient concentrations.

Day and flask number	Treatment	Phosphate (µM)	Nitrate (µM)	Ammonium (µM)	Silicate (µM)
D15 5	Co-culture	9.85	768.21	11.81	27.65
D15 7	Co-culture	11.44	943.09	12.83	23.40
D15 8	Co-culture	9.43	900.41	4.96	18.45
D15 18	Axenic	11.79	643.40	5.61	66.84
D15 20	Axenic	11.34	874.95	10.93	34.03
D15 21	Axenic	10.95	809.26	8.64	38.53
D20 10	Co-culture	15.90	810.91	19.48	65.17
D20 15	Co-culture	16.81	811.72	22.15	516.19
D20 17	Co-culture	11.37	750.14	0.63	63.49

Table S2. Annotation information for metabolites. Chemical shift values for candidate HSQC peaks are from Human Metabolome Databases (HMDB) except for DHPS, DMSP, homarine, and β-1,3-glucan (see Methods for details). Confirmed peaks are shown in bold. Peaks overlapping with those of other compounds are indicated by asterisks. Annotation confidence assignments are as follows: 1 = putative compounds with functional group information; 2 = partially matched to HSQC chemical shift information in the databases or literature; 3 = fully matched to HSQC chemical shift; 4 = fully matched to HSQC chemical shift and validated by HSQC-TOCSY; 5 = validated by a spiking experiment. Synthesis match indicates whether synthesis pathway genes were enriched in the diatom transcriptome, if known. Bacterial transporter match indicates whether uptake genes were enriched in the bacterial transcriptome at the same time point. Y = yes, N = no, nd = not determined due to no temporal difference/gene not known.

Compounds	1H (ppm)	13C (ppm)	Confidence level	Synthesis match	Bacterial transporter match
2,3-dihydroxypropane-1-sulfonate (DHPS)	**3.03, 3.11, 3.58, 3.69, 4.14**	**56.5, 56.5, 67.3, 67.3, 70.8**	4	Y	N
Dimethylsulfoniopropionate (DMSP)	**2.73, 2.91, 3.44**	**34.1, 27.8, 43.4**	4	Y	Y
Glycine	**3,55**	**44,3**	3	N	Y
Proline	**1.99, 2.07, 2.34, 3.32, 3.41, 4.13**	**26.5, 31.8, 31.7, 49, 49, 64**	4	N	nd
Glutamate	**2.09, 2.34, 3.74***	**29.8, 36.4, 57.6***	4	Y	nd
Glutamine	**2.12*, 2.44, 3.76***	**29.3*, 33.9, 57.2***	4	nd	nd
Alanine	**1.49, 3.78**	**19.0, 53.6**	4	nd	nd
Isoleucine	**0.93, 1, 1.25, 1.45, 1.96, 3.65***	**13.9, 17.4, 27.2, 27, 38.7, 62.5***	4	nd	nd
Valine	**0.98, 1.03, 2.26, 3.6**	**19.4, 20.8, 31.9, 63.3**	4	Y	nd
Glycine betaine	**3.25, 3.89***	**55.9, 68.6***	3	Y	N
Lysine	**1.43*, 1.49*, 1.72, 1.88*, 3.02, 3.75***	**24*, 24*, 29.2, 32.7*, 42.1, 57.5***	4	Y	nd
β-1,3-glucan	**3.51*, 3.51*, 3.56, 3.73*, 3.78, 3.92*, 4.79**	**70.9*, 78.4*, 76.0, 63.4*, 86.9, 63.4*, 105.4**	4	Y	N
Arginine	**1.68, 1.91, 3.24*, 3.76***	**26.5, 30.5, 43.3*, 57.3***	4	N	nd
Leucine	**0.94, 0.96, 1.7*, 1.71, 3.74***	**23.6, 24.8, 42.6*, 26.8, 56.2***	4	nd	nd
Acetate	**1,91**	**26,1**	3	nd	nd
Glycerol 3-phosphate	**3.61*, 3.68*, 3.78, 3.82, 3.86***	**65*, 65*, 67.6, 67.6, 74***	4	nd	N

Guanosine	**3.82***, **3.86***, **4.22***, **4.39***, **5.89**, **7.98***	**64.2***, **64.2***, **88.1***, **73.3***, **90.4**, **140.6***	4	nd	nd
Uridine	**3.8***, **3.91***, **4.12**, **4.23***, **4.35**, **5.89**, **5.9**, **7.86***	**63.6***, **63.6***, **87.1**, **72.1***, **76.5**, **105.1**, **92.1**, **144.6***	4	nd	nd
Aspartate	**2.71**, **2.8**, **3.91**	**39.3***, **39.5***, **55.1**	4	nd	Y
Glucose	3.23, 3.4, 3.46, 3.52, 3.7, 3.72, 3.81, 3.82, 3.89, 4.63, **5.22**	77, 72.5, 78.6, 74.3, 75.6, 63.5, 74.1, 63.3, 63.4, 98.7, **94.9**	2	Y	Y
Homarine	**4.37**, **7.98**, **8.04**, **8.55**, **8.72**	**49.3**, **130.2**, **129**, **149.4**, **148.6**	4	nd	nd
Phosphorylcholine	**3.2***, **3.57**, **4.15**	**56.5***, **68.9**, **60.6**	4	nd	nd
